# “In August, I Counted 24 Different Names”: Swedish Older Adults’ Experiences of Home Care

**DOI:** 10.1177/0733464820917568

**Published:** 2020-05-18

**Authors:** Marie Ernsth Bravell, Maria Bennich, Carola Walfridsson

**Affiliations:** 1 Jönköping University, Sweden; 2 Örebro University, Sweden; 3 Eksjö kommun, Sweden

**Keywords:** care coordination, community, decision making

## Abstract

In Sweden, many older adults receive care in their own homes. However, their perceptions of the experience of receiving home care has not been sufficiently examined. This study aimed to explore older adults’ experiences of receiving care and services in their homes and their perceptions of the care that they had received. In-depth interviews were conducted with 29 older adults. There were individual differences in their level of participation, but they mostly perceived their participation in the planning and provision of home care to be limited. Furthermore, their needs (and wishes), especially those that pertained to different aspects of time, were not always gratified. Organizational factors and care providers’ lack of competence and high levels of time pressure influenced their perceptions of care. It is important to consider the perspectives of older care recipients when developing home care.

## Introduction

Over the past 25 years, the old age care system in Sweden has undergone several important reforms, starting with the reform of ÄDEL in 1992, whereby the responsibility for the long-term care of older adults was transferred from county councils to municipalities. In 2008, the Swedish National Board of Health and Welfare (2008) reported that frail older adults are likely to benefit from a similar shift in home health care. In 2015, almost all regions in Sweden had implemented this reform. Therefore, the municipalities are responsible for the health care and social services that are provided in the homes of older adults. The consequent decrease in the number of beds and shorter stays in nursing homes (Schön et al., 2015) imply that municipal care is increasingly multifaceted. A wide range of home care services are provided to older adults with complex needs (Sandberg et al., 2019) to make it easier for them to continue living in their own homes (Szebehely & Trydegård, 2012). However, research findings on older adults’ perceptions of home care as limited.

## Home Care in Sweden

The needs of older adults are assessed to determine the level and type of home care that they require. Specially trained social workers assess and make decisions about older adults’ need for social services (e.g., assistance with self-care and performing activities of daily living), but it is the nursing assistants who provide these services. It is recommended that nursing assistants have an upper secondary (i.e., training in a vocational high school) or intermediate (i.e., junior college) education. Professional health care providers with higher educational qualifications (e.g., registered nurses with a 3-year university/college degree) make decisions about and sometimes provide health care services (e.g., treating wounds and administering prescribed drugs). Providing home care to older adults includes several tasks that can range from making a quick visit (e.g., checkup and delivering Meals-on-Wheels) to providing comprehensive health and social care (Sandberg et al., 2019). One of the impetuses for the home care reform was the need for better coordination of resources and collaboration between social service and health care providers (National Board of Health and Welfare, 2008).

## Participation of Care Recipients in Home Care

Social service and health care providers share the common goal of participation and involvement of the care recipient in home care, often guided by Arnstein’s (1969) participation ladder. The underlying principle is that care recipients should play a key role in managing their own health such as by making important decisions and setting goals (Longtin et al., 2010). In Sweden, participation and involvement are regulated by law (Swedish Patient Law, 2014). The National Board of Health and Welfare (2009) has emphasized that older adults should be treated in accordance with their social contexts and with due consideration to their unique circumstances and needs. Involvement in care and service has also been found to have positive effects on health status, self-care activities (Näsström et al., 2015), and safety (Martin & Larsen, 2012). Older adults appear to want to be involved in their own care and have underscored the significance of sharing a caring relationship with their health care providers but research on care recipients’ perceptions of involvement in home care is limited (Boström et al., 2017; Lyttle & Ryan, 2010).

This study aimed to examine older adults’ experiences and perceptions of receiving care and services in their homes.

## Method

### Design

This study was a part of a larger study on the effect of home care reform in a region in the southern part of Sweden. In the parent study, questionnaires were administered to home care staff, and data were collected from different sources and analyzed (Ernsth Bravell, 2015). In this study, qualitative interviews were conducted with older adults who had been receiving health care and social services in their homes. The interviews were conducted by specially trained nurses. Qualitative content analysis was used.

### Participants and Procedures

The participants were recruited from 13 municipalities in a medium-sized region in southern Sweden. Participants who met the following criteria were identified with the aid of a designated contact person in each municipality: the older adult should have been enrolled in both *home care* and *social services* for at least 6 months and be able to participate in an in-depth interview (i.e., cognitive and language skills). A designated contact person (i.e., a registered nurse or social worker) in each municipality identified and contacted individuals who met the inclusion criteria. They provided both oral and written information about the study and asked them if they could provide their contact information to the project manager. Consenting individuals were contacted by telephone; again, they were provided with information about the study, and they were asked to indicate if they would be willing to participate in the study. Prior to the interview, the participants signed an informed consent form.

Out of 34 identified individuals, 29 consenting older adults, who represented all the 13 municipalities in the region, were interviewed. Their mean age was 79 (*Mdn* = 80, range = 66–91); a majority (i.e., 21) of them were living alone, and 20 of them were women. On average, they have been enrolled in home care for 2.5 years. They lived in both rural and urban areas. Four, 13, and 12 participants lived in a municipality with approximately 1,40,000 inhabitants, between 15,000 and 50,000 inhabitants, and less than 15,000 inhabitants, respectively.

A majority of the interviews were conducted in older adults’ homes (one interview was conducted via a telephone). Semistructured interviews were conducted using an interview guide that focused on participants’ experiences of receiving care and support in their own homes. Open-ended prompts and questions such as “Tell us about your experience of receiving home care” and “What is important to you when you receive care in your home?” were used. The interviews lasted for approximately 58 minutes (range = 25–90) and were audiotaped and transcribed verbatim. Prior to analysis, each participant was assigned a code, which included an alphabet and a number. Under “Results,” these codes as well as the gender and age are presented alongside selected quotes.

### Analysis

The data were subjected to qualitative content analysis. Data analysis proceeded from a focus on the general to the specific. This was achieved by means of open coding, the creation of categories, and abstraction (Elo & Kyngäs, 2008). With regard to abstraction, text condensation was achieved through additional critical discussions among the researchers, and the process yielded eight subcategories and three categories. The notes that were written in the margins were entered into a coding scheme and, thereafter, grouped and labeled with descriptive codes. All the authors read the transcripts and conducted the initial analysis from their unique perspectives (i.e., gerontology, social work, and practice). Discussions were conducted until we reached an agreement about the labeling of codes. The condensing process was subjected to further critical discussion within the research group, resulting in abstraction to eight subcategories and three categories. Interviews were conducted in Swedish, and the quotes that have been included in this article were translated by the authors.

### Ethical Considerations

This study adhered to the ethical principles of the Declaration of Helsinki, and the approval and permission to conduct this study were granted by the Regional Ethical Review Board, Linköping (dnr 2012/22-31).

## Results

**Figure 1.**
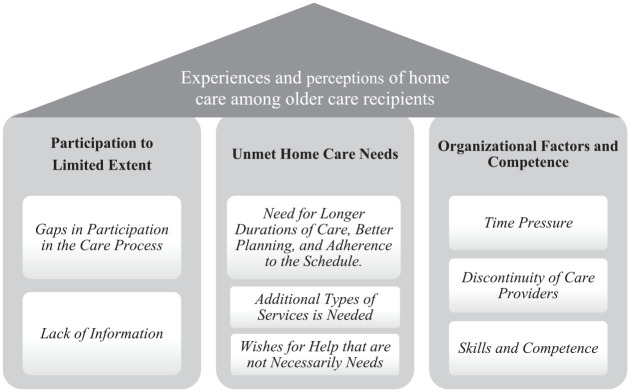
Illustration of the three categories (*a*) *participation to limited extent*, (b) *unmet home care needs*, and (c) *organizational factors and competence*, that emerged from the analysis of the participants’ experiences of home care.

Content analysis yielded the following three categories, which captured older adults’ experiences and perceptions of the care that they had been receiving in their homes: (a) participation to limited extent, (b) unmet home care needs, and (c) organizational factors and competence (see Figure 1). Despite individual differences, participants reported a relative lack of participation in planning, performance, and follow-up home care. A lack of participation can cause frustration, which was subsumed under the category, “unmet home care needs.” Specifically, the participants perceived a gap between the home care that they had been receiving and their perceived needs and wishes. Furthermore, the participants were frustrated but also ascribed importance to organizational factors and the competence of the staff.

### Participation to Limited Extent

There were individual differences in older adults’ levels of participation and knowledge about the care process, as well as opportunities for participation (i.e., as per the Patient Law). The interviews revealed that the participants found it difficult to understand their role in care planning. They had sometimes made inappropriate decisions because they had not received adequate information.

#### Gaps in participation in the care process

Overall, the participants reported that they were not involved in the initiation of care. In some cases, the initiation of care had occurred in a hospital, following an unexpected injury or disease or planned surgery, and the participants did not feel as though they were involved in this process.

We had a meeting . . . at . . . at the hospital . . . and there, I met the ladies who took care of the management of the home help services . . . And, I guess, a preliminary plan was set that included that I would get some help at home . . . So it started as I remember it. (H1, man, 91 years)

Furthermore, the participants reported that they were not involved in the planning of care. Difficulties in formulating requests for care and services may result from a lack of information about the purpose of the meeting (for planning) and the legal rights of the participants. A common comment concerning their participation in the planning of care was as follows: “No, no, no . . . It [home care] just came” (D1, woman, 87 years). This indicates that the participants had not been informed about their right to influence the planning of care.

With regard to the actual provision of care, older adults reported that they had tried to influence it albeit with varying results. They reported that they had tried to influence their care schedules. They also reported trying to influence the actual performance of tasks and the specific manner in which different tasks were performed to some extent. They also reported that they had tried to influence the support that they had received for engaging in daily activities (e.g., cleaning, cooking, and doing the laundry), but their influence on health care (e.g., wound dressing and injections) was to a much lower degree. They further observed that there may have been some kind of follow-up, but participation in follow-up care varied considerably. Indeed, their comments ranged from “yes, the . . . a paper came that we filled in . . . ” (M1, woman, 88 years) to “no, no, there has been no such thing” (D1, woman, 87 years).

#### Lack of information

The participants considered information about the care process to be important, even crucial, to their own possibility to participate. However, they also considered that they were not provided with adequate information (e.g., how to apply for less or more care): “I need more help . . . or at least want it . . . but I have not applied for it. I do not know how it works.” (M2, man, 80 years).

Furthermore, the staff did not understand and address the importance that they ascribed to information. Often, the need for care had emerged unexpectedly. To be able to influence their daily lives, it was important to them to have information about their care providers (i.e., organization) and the care process.

I want to be like any other senior . . . I am not an invalid. This is just something that happened, and, if I am now supposed to handle my life with joy . . . which is what I want . . . it would be better with more information. (A1 women, 78 years)

Although participants differed in the degree to which and contexts in which they wished to participate, it was something that they strived toward, and wanted information about.

### Unmet Home Care Needs

There was a gap between the care that the participants had received and their experienced need for support. The participants were satisfied with several aspects of the home care that they had been receiving. However, because many of their needs had not been satisfactorily gratified, they were unable to lead the lives that they had been living prior to their illness or injury. For example, their need for longer durations of help (minutes per visit) and support to perform certain tasks, which the community care did not offer, was not fulfilled. Many participants reported that they *wished* for help with specific tasks, even though it may not be considered to be a *need*.

#### Need for longer durations of care, better planning, and adherence to the schedule

Participants wished to receive longer durations of care (minutes per visit), but this need was not satisfactorily fulfilled. They felt that cleaning and cooking could not be satisfactorily accomplished within the duration of time that they were afforded (i.e., not in the manner in which they accomplished it prior to their illness or injury). Some participants attributed their perceptions of shorter durations of care (including both minutes per, and number of, visits) to their loneliness and insecurity.

But, by that, they thought I didn’t need to have another visit. And, I miss it very much because I’m alone here from the last visit. (A1, woman, 78 years)

Some participants reported that they needed more minutes per visits and also more frequent visits because of their medical conditions (e.g., diabetes). The additional time and visits were also related to a sense of security.

And then, it has been important to have an extra snack in the evening. (F2, man, 85 years)

Their desire for greater control over their time remained unmet (e.g., the time at which home care and social service providers were asked to arrive). Many participants mentioned the specific time at which they wished to wake up and get out of bed in the morning and the time at which they wished to go to bed at night. Specifically, they wanted to wake up early and go to bed at a later time. However, the schedule of the care providers did not permit them to receive help at the desired time.

The worst thing is that I want to sit up late and watch TV late in the night (laughing), but then, I need help to get to bed, and it will be difficult. (J1, woman, 84 years)

The participants also expressed a lack of control over their schedules. Specifically, they could not be sure that the staff would arrive at the stipulated time. They also had to wait for a long time for the staff to arrive in the morning or at night. Consequently, because the care providers arrived at different times each day, the care recipients had limited control over the daily schedules.

#### Additional types of services needed

Although the participants were aware that they could not get help with all their needs, they expressed a need for help with tasks that were important to them (e.g., help with ironing and pressing). They had ironed and pressed their laundry all their lives; however, currently, they were unable to independently accomplish such tasks, and they were disappointed that the municipality did not help them with such tasks.

I want my laundry pressed and ironed, or no . . . not ironed but pressed . . . but they don’t do that. (D1, woman, 87 years)

Participants also required help with moving the lawn and shoveling snow. These tasks are not related to personal or medical care and are not necessary to manage one’s daily life. Therefore, assistance with such tasks is not proved as a part of public care services.

You have received a paper from the municipality; it is clear that “we do not cut the grass anymore. (F5, woman, 80 years)

#### Wishes for help that are not necessarily needs

Many participants were aware that some of their expressed desires for additional services were *wishes*, not *needs*. However, their wishes (e.g., regarding food) were important to them. They were, for example, unhappy with the food that the municipality had been sending them and wished for different kinds of dishes and food items.

Yeah, well . . . it’s not exactly what I’m looking forward to, in the municipality’s food. (M1, woman, 88 years)

They also wished to receive cooked meals rather than meals that were served in a box and required heating. However, they were aware that municipalities had their own schedules and that they had to make compromises, even if it meant that their wishes would not be met. The participants also *wished* for longer durations of care (minutes per visit) and additional help, but they were not sure if they really *needed* it (i.e., if it could be considered a need).

I want longer showers, which are properly done. But, there isn’t time. (D3, woman, 80 years)

### Organizational Factors and Competence

The participants’ experiences of home care were inextricably linked to organizational shortcomings, namely, the working conditions and prerequisites of the home care staff. In particular, they mentioned a shortage of time, continuity, and flexible schedules for the home care provider. The following issue was also identified: the care providers’ skills and competence.

#### Time pressure

With regard to time pressure, the participants often reported that the care providers were working under harsh and strained conditions. For example, the staff did not have time to sit down and talk to the participants. The participants reported that the staff only had a few spare moments to talk to them. They also believed that time pressures had increased over time.

They are far too rushed [now]. Yes, before I could serve them coffee and a cinnamon bun before they started cleaning. Now, they just don’t have time for it. (E1, man, 88 years)

Nevertheless, the participants demonstrated some understanding of the organizational conditions. Therefore, they were empathetic toward the care providers and did not blame them Instead, they focused on the organization and those who organized care work. Some participants mentioned that care managers were responsible for the care providers’ work schedules.

They are in a hurry, and they work. Well, they have their managers and responsibilities for many patients; so, it’s not always that easy. (F1, woman, 88 years)

The participants also stated care providers’ limited control over their schedules and responsibilities (i.e., who should do what and when). They were concerned about the future and worried that the situation may become worse.

But now, they have changed the schedule again; so, now, it falters. I don’t know how it will develop. (J2, woman, 87 years)

#### Discontinuity of care providers

The participants reported that care planning sometimes has flaws and that it is difficult to maintain the continuity of care providers who visit their homes and provide care. Schedules changed often, and it was not unusual for them to receive care from several new or temporary workers who were unaware of their routines and habits. It was particularly problematic when they received care from several care providers.

Yes, that becomes troublesome when they change the times too often. Yes, and also when they are too many. (J2, woman, 87 years)

The discontinuity of care providers was particularly evident during vacation periods. Indeed, several participants reported that this was particularly prominent during summer. Then their care providers changed regularly (e.g., each day or each slot).

In August, I counted 24 different names. (F4, man, 96 years)

In this regard, it is noteworthy that the older adults considered the care providers who worked as summer employees to be neither particularly experienced nor well trained.

#### Skills and competence

The participants believed that it was important for all care providers, not only summer employees, to be well trained and experienced. The participants ascribed importance to the formal education of care providers. Furthermore, they believed that some shortcomings of the care providers were attributable to their limited skills and competence. They preferred to receive care from providers who had received formal education and training. They believed that formally trained care providers are likely to provide better care. Therefore, receiving care from such providers enhanced their sense of security.

An absolute requirement is an auxiliary nursing education, because that is too often lacking; there are too many without [it]. (I2, man, 69 years)

With regard to the ability to perform daily tasks, care providers sometimes lacked the ability to lift and maneuver older adults with mobility problems (i.e., ergonomics and locomotion). The participants reported that these problems, which are attributable to a lack of skills and inadequate education, were particularly experienced during summers, when regular care providers were replaced by summer employees. They also had difficulties performing simple tasks such as cleaning and washing. The participants sometimes had to instruct the summer employees about how certain tasks ought to be performed.

Some of them, and especially in the summer, and because of vacations and that, are incompetent schoolgirls, and I don’t think that is good; it isn’t how it’s supposed to be. (E1, man, 88 years)

The participants indicated that inexperienced and untrained care providers contributed to a sense of insecurity among the participants. Different participants appraised the relational style of their care providers differently. However, the emergent findings underscored the importance of the manner in which care providers interact with older adults.

Yes, they [care provider] are supposed to be nice. But everyone who comes here is good. It is as if all of them had been handpicked. (K1, woman, 80 years)

In contrast, some participants reported that their care providers sometimes failed to introduce themselves or take initiatives. In other words, they lacked basic etiquette. The participants perceived this to be indicative of a lack of competence and skills.

The worst thing, for me, is when people come in and don’t make a sound and just stand there. That is what I think is the most important—that they say who they are. (L2, man, 69 years)

## Discussion

The starting point of this study was older adults’ perceptions of the care and services that they had been receiving in their homes. The interviews revealed that older adults participated in care processes to limited extent, had unmet needs, and were aware that organizational factors greatly influenced the care and services that they received. All the content categories included a subcategory that pertained to time; the specific aspects of time that was addressed varied depending on the content category under which it was classified. Older adults wanted to and had tried to influence the duration of time (minutes per visit or numbers of visits) for which they received care. They reported that the duration of time for which they had been receiving care was unsatisfactory. Furthermore, they reported that limited time (too few minutes scheduled per visit or too few visits) was one of the shortcomings of organizations that provided them with care. This was evident from the high levels of stress that were experienced by the care providers. This is an important explanation for older adults’ evident frustration regarding a lack of time and limited control over their time.

The participants reported varying patterns of participation, but most of them reported a lack of participation, especially in initiating and planning care. They did not understand their role in and their right to influence their care. According to the Swedish Patient Law (2014:821), patients have the right to influence the planning and provision of care and participate in their care. This requires care recipients to gain the necessary information (Arnstein, 1969) and feel safe about their collaboration with various professionals (Bennich, 2012). Our participants reported a lack of both participation and sufficient information, especially with regard to help with performing daily activities. Many participants had sustained injuries or contracted diseases. They were in a vulnerable state and may have had difficulties coping with and adapting to their new circumstances. Past studies have found that care recipients in similar circumstances do not play a role in the process of making decisions about their home care; social workers inform them about what is available, rather than asking them about their needs (Janlöv et al., 2006). Older adults’ knowledge about the public home care system is largely founded upon their initial encounters with their care managers (Olaison, 2009). It is important to pay attention to the mental status of frail older adults during the needs assessment and ensure that they adequately understand the information that they have been provided with and encourage their participation in their care. Otherwise, they may experience feelings of insecurity (Boström et al., 2017) and dissatisfaction (National Board of Health and Welfare, 2019) and, as seen in this study, a lack of participation.

A lack of participation was also reflected in the contents of the category that pertained to unmet needs. For example, the participants were unsure if their expectations were a wish or a need; indeed, it is difficult to differentiate between the two. As mentioned earlier, the provision of home care to older adults is consistent with not only the Social Service Act and Health Care Act but also the Patient Law. In 2015, this law was revised (2014:821) which aimed to strengthen patient participation by clarifying their role and upholding their integrity. However, a recent evaluation (Vårdanalys, 2017) showed that municipal health care providers receive insufficient support for implementation. Older adults’ participation has not increased in recent years. Instead, it has declined further, and this trend is consistent with the present findings. Jarling et al. (2018) identified similar gaps between older adults’ legal rights, their experiences of receiving home care, and their ability to influence it. The present findings reveal that older adults’ wishes and needs are not unreasonable. By encouraging their participation in planning, execution, and follow-up, home care can be improved. However, as Breitholtz et al. (2013) have contended, decision-making is not a simple task and requires one to maintain an act of balance that can be difficult to achieve. As older adults become more dependent on caregivers’ help, the opportunities to be independent reduce, and this is stressful to them. There is a need to understand older adults’ daily lives to provide them with greater opportunities for independence and encourage participation. Based on the present findings, a relational approach toward the promotion of autonomy rather than the traditional individualistic and person-centered approach is recommended.

According to the participants, their care providers were often working under challenging organizational conditions. This raises questions about work environments. Our participants mentioned that it is the manager who makes decisions about schedules and tasks. Lundgren et al. (2015) found that leadership plays an important role in care providers’ experience of their work environments. Within the context of home care, there is a physical distance between leaders and care providers, with limited opportunity for interactions between care providers and leaders (Bennich, 2015; Lundgren et al., 2015). However, care providers’ work environments and satisfaction levels do affect older adults’ perceptions of care (Lundgren et al., 2018), so it is necessary to develop the working conditions in the home care. Another issue, to some extent related to the working conditions, is the discontinuity of care providers. This was expressed as a concern for the participants but also as a worry for the future. The National Board of Health and Welfare (2019) confirm this concern; they found an increase in discontinuity of care providers during the years of 2007 to 2017.

When receiving long-term community care, it is important for older adults to feel safe and secure (De Sao José et al., 2016) and for care providers to have undergone adequate training and possess the necessary skills (Bennich, 2015). Older adults feel secure when trained care providers are accessible around the clock (Boström et al., 2017) and they also consider safe and secure care to be inextricably linked to relational aspects (Bennich, 2012). In our study, some participants reported that their home care providers lacked adequate training and competence in providing treatments. There is a distinction between *formal* and *actual competence* (Ellström, 1997). Formal competence is the human capital that care providers develop through training and education. Actual competence, in contrast, is one’s capacity to successfully handle a certain situation or complete a certain task. The competence of care providers is related to the care that they provide, and it includes their ability to perform practical tasks and treat older adults appropriately and with respect; this is a prerequisite for the development of reliable relationships. Approximately 30% of the care providers were assistants who had not received any formal health care training, and community care is increasingly provided to frail older adults with complex health problems (Bing-Jonsson et al., 2016; National Board of Health and Welfare, 2019). The participants of this study emphasized their preference for care providers with formal and actual competence. This poses a significant challenge to the care of older adults in the future. Summer employees are necessary; however, it is important for new care providers to receive adequate training and practice during the summer and other vacation periods. It is also of the utmost importance for organizations to invest in the adequate training of new and temporary staff. For example, the interviews revealed that it is necessary for new and temporary care providers to learn the correct method of lifting older adults. This is primarily a question that pertains to organizational resources and leadership (Kjellström & Sjölander, 2014; Lundgren et al., 2015), but care providers most frequently interact with older adults and are therefore likely to provide optimal care.

### Limitations and Strengths

These results were derived from a substantially large data set that was generated through interviews that were conducted with 29 older adults who varied in their gender, age, and living conditions. The study was a part of a larger study (Ernsth Bravell, 2015), and the analyses of the interviews were secondary, based on already collected data. This can be regarded as a limitation of this study because the approaches using which the data could be analyzed were limited, and it was not possible to ask follow-up questions to gain a deeper understanding of participants’ feelings or the phenomenon of interest. Therefore, to address the research question, we conducted qualitative content analysis in accordance with the procedure that has been described by Elo and Kyngäs (2008). The study lacks information about participants’ medical conditions and availability of informal caregiving, and it excluded persons with cognitive impairment, a large portion of people who receive home care. The findings must be considered within these limitations.

The study was conducted in a region in southern Sweden, including one large (i.e., one of the 10th largest municipality in Sweden), one medium-sized, and 11 small municipalities; therefore, the findings may not be generalized to older adults from large urban municipalities.

### Implications for Research and Practice

In Sweden, patients have the legal right to participate in their care process. However, the present findings underscore the need for care organizations to implement the tools that facilitate participation (e.g., relevant information about the care process and organization). More importantly, staff policies should require home care providers to regularly undergo competence development programs. Furthermore, they should be motivated to establish good relationships with older adults, and the continuity of care providers should be promoted. Even though this study was conducted in only one region in Sweden, care in the home of older adults is encouraged in most Western countries. Therefore, the participation of older adults and establishment of good relationships between care providers and older adults should be promoted in other contexts as well. A more comprehensive picture of home care may be received by including care workers and family members in future research.

## Conclusion

An examination of older adults’ experiences of receiving home care revealed that their level of participation in the care process varied considerably and was mostly perceived to be limited. This was also reflected in their responses that pertained to their unmet needs. Organizational factors and the competence of the care providers influenced their experiences of care. Home care organizations should use these findings to develop new policies and implement tools that encourage participation, nurture the competence of care providers, and promote staff continuity.
